# Quality improvement for self-management: the DIALOG+QI framework

**DOI:** 10.1136/bmjoq-2025-003812

**Published:** 2026-01-22

**Authors:** Pedro Delgado, Carlos Santos, Lenna Adley, Amar Shah

**Affiliations:** 1 Institute for Healthcare Improvement, Boston, Massachusetts, USA; 2 Harvard T H Chan School of Public Health, Boston, Massachusetts, USA; 3 Quality Improvement Department, East London NHS Foundation Trust, London, UK; 4 Institute of Psychiatry, Psychology & Neuroscience Library, King’s College London, London, UK; 5 East London NHS Foundation Trust, London, UK; 6 NHS England, Redditch, UK

**Keywords:** Quality improvement, Chronic disease management, Long-Term Care, Mental health, Self Care

## Introduction

### ‘They cannot argue with what they see’

 For 20 years, LA tried to be heard. She repeatedly told healthcare professionals that something specific was wrong, that her diagnosis did not fit her lived experience and that she needed different help. Instead, she was told she was not accepting her diagnosis. The consequences were devastating: 3 years in secure hospital settings costing the NHS thousands of pounds, suicide attempts requiring intensive care, friends being told to consider turning off her life support. “Had I been listened to 20 years earlier,” she reflects, “I wouldn’t have had the last 20 years robbed from me. It almost cost me my life.”

The breakthrough came through an unexpected route. While involved in People Participation at East London NHS Foundation Trust, LA attended an introductory quality improvement (QI) session. The basic tools—driver diagrams, baseline measurement and run charts—sparked a realisation: “If we can use these tools in our work, why can’t I use them to take control of my care?” Using post-it notes, she mapped everything she was struggling with, grouped similar symptoms, created a driver diagram and then developed scoring systems to track patterns over several months.

The data revealed what 20 years of speaking could not convey. Every chart showed the same unmistakable pattern: dramatic symptom escalation 2 weeks before her period, sustained severity, then rapid improvement days afterwards. When she took this evidence to her GP, everything changed. “He couldn’t argue with the figures and the literal pattern,” she explains. The hospital confirmed what she had known for years: premenstrual dysphoric disorder—a particularly challenging condition to diagnose.^1^ With the right treatment, her mental health and well-being reached levels she had never experienced. She secured her first full-time job.

LA’s insight captures a fundamental tension in modern healthcare: they can argue with what they listen to, but they cannot argue with what they see. QI tools transformed her mode of communication from speaking—which could be dismissed—to showing—which could not.

This challenge extends beyond patients. The nurse expertly applying PDSA cycles to improve her ward may feel powerless against her own burnout. The physician championing evidence-based practice may tackle stress without systematic methods. As the Institute for Healthcare Improvement’s Framework for Improving Joy in Work recognises,^2^ supporting healthcare workers’ well-being is essential for both retention and quality of care.

LA’s story represents a broader pattern among staff, patients and citizens: individuals extending continuous quality improvement (CQI) methods (the systematic approach to testing and implementing changes) into self-management. This is an untapped frontier for health improvement and deeper partnership, yet these innovations remain anecdotal: no structured framework supports them. We propose DIALOG+QI as that framework. The same approaches transforming clinical systems^3^,^5^ could bridge the gap between knowing what to do and learning how to do it reliably, in real life.

## The self-management chasm

The case for effective self-management in chronic conditions—particularly mental health—is uncontested.^6^,^8^ Robust evidence demonstrates that empowerment interventions improve patient outcomes across long-term conditions, with measurable gains in quality of life, self-efficacy and psychological well-being.^6 9 10^ Yet a chasm persists between knowing what might help and developing the personal insight and agency to determine what works for oneself, when it works and how to adapt when circumstances shift.^9^

LA’s experience illuminates this chasm with painful clarity. She received excellent care: evidence-based treatments, multiple therapeutic approaches and specialist consultations. She tried ‘nearly every antipsychotic, every antidepressant in the book’, mood stabilisers, cognitive behavioural therapy (CBT), dialectical behaviour therapy (DBT) and counselling—the full range of available interventions. The problem was not the absence of knowledge or resources. The problem was that *the only person who had all the pieces was not being listened to*.

Healthcare systems excel at specialisation—cardiology, psychiatry, nutrition and physiotherapy—each domain optimised through rigorous QI. But as LA observed, “all these different departments stay in their own lane, afraid to go into somebody else’s lane because that’s not their speciality. By doing that, they’re not seeing the whole person.” We asked who holds all the pieces in fragmented care. “It’s you,” she said simply. “But the person doesn’t get listened to.”

This paradox sits at the heart of modern healthcare’s relationship with self-management. The patient is simultaneously the only one with complete information about their lived experience across all domains AND the least empowered to act on that holistic understanding. The person experiences the synthesis but lacks tools to make that synthesis visible, actionable and shareable.

This is the *self-management chasm*—not lacking information but lacking structured approaches to transform generic guidance into personalised, actionable wisdom. Healthcare often positions individuals as passive recipients of expert knowledge, when we should be honouring their agency, helping them become expert observers and systematic improvers of their own lived experience.^9 11^

Why has CQI not been widely applied at individual self-management level? Hard to know for sure. Traditionally, CQI has been conceived as a team sport, reliant on shared data and collaborative testing.^3^,^512 13^ Yet if we reconceptualise each person’s life as an interconnected personal system—much like the clinical microsystems we aim to improve^3^,^5^—then CQI’s core tools and concepts become potentially transformative for personal health maintenance, recovery and improvement.

## DIALOG+: a framework for improving quality of life

Existing patient-centred approaches offer glimpses of this potential. One example is DIALOG+, a structured intervention designed by Priebe *et al*.^14^ Implemented across the UK and internationally, DIALOG+ structures clinical encounters around patient-rated satisfaction with quality of life and treatment domains, prompting cocreation of practical actions revisited at each meeting. Recent studies demonstrate DIALOG+’s effectiveness across diverse settings from chronic depression and psychosis in secondary care to chronic physical conditions in primary care, with high acceptability among both service users and clinicians.^14 15^

**Figure 1:**
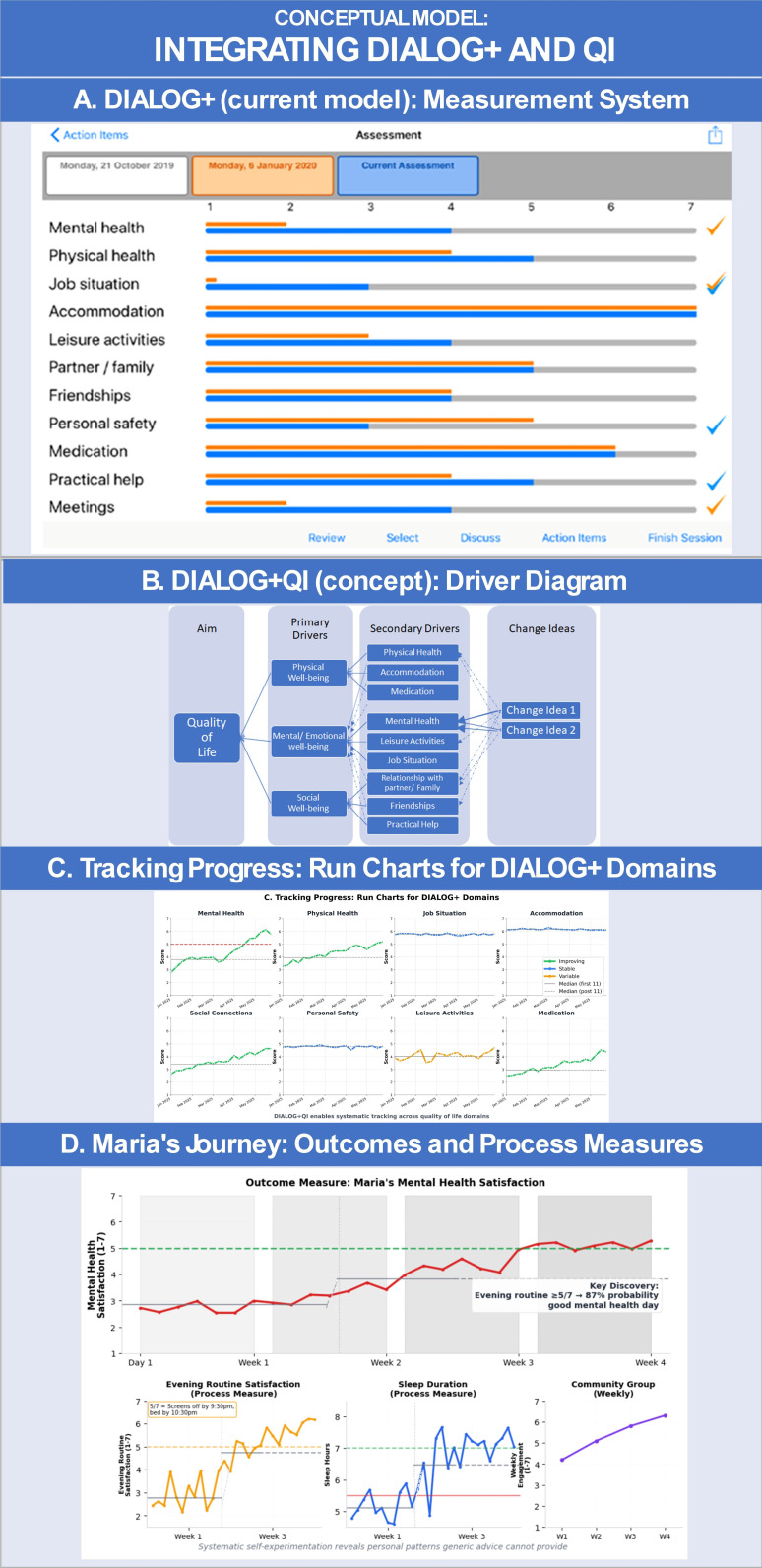
Conceptual model for integrating *DIALOG+QI*. (A) DIALOG+ (current practice): routine outcome measurement across life and care domains. (B) *Driver diagram illustrating how DIALOG+ domains structure improvement efforts through primary and secondary drivers, linked to targeted change ideas.* (C) *Example run charts tracking progress in individual DIALOG+ domains over time.* (D) *Visualisation of an individual patient’s journey (Maria), combining outcome and process measures to monitor improvement in real-world practice.* DIALOG+QI, DIALOG+ and quality improvement*.*

DIALOG+ embodies several CQI principles: baseline assessment,^12^ routine measurement,^16^ patient-led priorities^3^ and iterative improvement conversations.^2 5^ The domains it explores—mental health, physical health, job situation, accommodation, leisure activities, relationships, personal safety, medication, practical help and overall well-being—provide comprehensive coverage of life areas affecting health and recovery. Though its current data feature just a snapshot comparison with a recent past score (figure 1A) rather than tracking change over time, the foundation for meaningful improvement is already present.

LA valued DIALOG+ sessions with her mental health team—but the sessions were periodic touchpoints, with no infrastructure to extend that systematic thinking into her daily life between encounters. This gap between clinical conversations and daily self-management pointed towards an enhanced approach that could fully embrace personal continuous improvement.

## Envisioning DIALOG+QI: making personal CQI tangible

Building on the empirically validated DIALOG+, we envisage DIALOG+QI—a framework to bring well-established QI methods into individual health self-management. Where DIALOG+ creates structured clinical conversations about well-being across multiple life domains, DIALOG+QI can extend that thinking towards continuous improvement.

The core proposition is straightforward: the fundamental tools of organisational QI—driver diagrams for mapping complexity, baseline measurement and run charts for revealing patterns and PDSA cycles for testing changes—can be meaningfully adapted for personal health management. This is not about turning individuals into QI professionals or imposing technical methodologies onto personal life. Rather, it is about democratising the systematic thinking that can transform people’s relationship with their own health. Continuous improvement transcending organisational boundaries.

Imagine Maria, a 29-year-old retail manager, 6 months into her recovery journey. Working with her peer support worker using an enhanced DIALOG+QI approach. They cocreated a personal driver diagram (figure 1B) linking her aim—“Within 4 weeks, increase days where I rate my anxiety satisfaction as 5/7 or higher, from 2 days to 5 days per week, so I can feel good about myself again”—to factors Maria believes she can influence: better sleep, meaningful social connection and regular physical activity. She hypothesised that ‘consistent evening routine’ (driver for better sleep) and ‘attend community group one time a week’ (for social connection) will help.

Maria tests whether a consistent evening routine improves sleep. Using her personal dashboard (figure 1D), she tracks daily anxiety (outcome measure) alongside ‘screen-off by 21:00’ and ‘sleep hours’ (process measures). After 3 weeks, personal run charts reveal a clear pattern: consistent evening routines (screen-free+good hours of sleep) correlate with more stable, lower anxiety levels.

While LA’s story demonstrates the framework’s diagnostic power—revealing patterns invisible to clinical observation over 20 years—Maria’s journey illustrates its everyday application: turning generic guidance into personalised, actionable insight. Both illuminate the same principle: systematic self-observation generates evidence that transforms both personal understanding and clinical collaboration.

The enhanced DIALOG+QI framework (figure 1) would provide the following.

- *Personal driver diagrams* mapping individual aims into practical, changeable factors.^12 17^
- *Personal run charts* visualising progress and patterns over time.^16 18^
- *Mini-PDSA dashboard* where every practical step becomes something individuals can systematically test, monitor and refine^12 14^ (see online supplemental file 1 for detailed visualisation).

This framework emerged from practice. Three interconnected sources inform the DIALOG+QI concept, with lived experience as the foundation.

1. *Systematic personal QI practice already exists*: the conceptual breakthrough came through conversations with LA, whose approach to personal QI demonstrated all the hallmarks of rigorous improvement work: baseline measurement enabling pattern recognition, data visualisation facilitating clinical collaboration, personal discovery leading to targeted interventions and measurable outcomes, including *improved health* and *return to employment*. Critically, LA was not unique. As we explored further, similar narratives emerged—people systematically tracking well-being, creating driver diagrams of personal health challenges, running small tests of change in their daily lives and using PDSA cycles to refine self-management strategies—but these innovations remain largely anecdotal.

2. *DIALOG+ implementation revealed the gap*: since 2014, East London National Health Service (NHS) Foundation Trust has been implementing DIALOG+ across mental health services.^14 15^ While patients benefit enormously from structured clinical conversations using DIALOG+, sustaining self-management beyond the clinical encounter goes beyond the intervention’s remit: DIALOG+ is not equipped with systematic means to test what works for patients personally, to track patterns across the multiple domains DIALOG+ explores or to adapt strategies when circumstances change. LA’s innovation was recognising that the same systematic thinking she was learning through QI could extend DIALOG+’s structured approach into the spaces between appointments. The domains DIALOG+ helped her articulate became the starting points for her driver diagram. The collaborative review process became enriched by evidence she brought: “I was able to take that chart to my GP, and he said, ‘Yeah, there’s quite an obvious pattern here.’ My mental health team then worked closely and collaboratively with me, alongside the physical health side.”

3. *Theoretical alignment explains why it works*: the framework aligns with established principles that help explain why LA’s approach succeeded where years of conventional treatment struggled.

- *Experiential learning theory*^19^ emphasises that adults learn most effectively through concrete experience, reflective observation, abstract conceptualisation and active experimentation—precisely the cycle QI tools facilitate.
- *Patient activation theory*^9^ suggests that individuals progress through stages from passive recipients to active partners in health management. QI tools accelerate this progression by providing structured approaches that build confidence and competence systematically. People move from passive recipients to become active investigators of their own health, generating evidence that demands professional response.
- *Self-determination theory*^20^ identifies autonomy, competence and relatedness as fundamental psychological needs driving sustained behaviour change. QI tools support autonomy (individuals decide what to measure and when), build competence (developing skills in data collection and interpretation) and strengthen relatedness (evidence enables more collaborative relationships with clinicians). *LA’s experience demonstrates all three*: she chose her measurements, developed her capabilities and transformed her clinical relationships.

## Safety considerations

While DIALOG+QI offers promise, certain contexts require personal QI approaches to be developed with particular care.

### Measurement burden and its impact

Excessive tracking can create anxiety, obsessive behaviours or counterproductive self-monitoring. The framework should emphasise collecting ‘just enough data to drive learning’—no more, no less.^12 16 18^ Some individuals will thrive with detailed monitoring; others will prefer focusing on one or two key areas. Measurement should always serve learning, not become an end in itself. Clear guidance is needed on when to measure, how much to measure and when to stop. For people with mental health conditions, measurement approaches require careful calibration. The framework cannot be applied universally without clinical judgement—people experiencing acute mental health crises, significant cognitive impairment or severe health conditions will require clinical assessment before introducing structured self-monitoring approaches. LA’s success came through finding the right balance for her circumstances at the right time in her recovery journey.

### Clinical integration and appropriate escalation

LA’s story demonstrates both the power and the limits of personal QI. Her systematic data collection enabled crucial diagnosis and treatment—but that diagnosis and treatment still required professional healthcare. The framework is not a substitute for clinical care; it is a complement that can make clinical care more effective. Warning signs requiring clinical attention (worsening symptoms, suicidal ideation and severe distress) need to be explicitly communicated. The framework can help people recognise when patterns revealed through personal QI indicate the need for clinical escalation rather than continued self-management experimentation. These boundaries apply to all self-management approaches: the framework’s role is to make them more visible and actionable.

### Access, equity and individual responsibility

While technological tools could support DIALOG+QI implementation, the framework needs to remain available to those with limited digital access or literacy. LA’s approach was essentially analogue—Post-it notes, paper charts and manual scoring. Paper-based approaches and appropriate support should be available. As LA noted, accessibility means meeting people where they are: videos for those who struggle with reading, written materials for those who prefer text and in-person support for those who need collaborative introduction to the tools.

Perhaps the subtlest risk is emphasising individual capability to improve one’s own health, inadvertently reinforcing narratives that health is purely an individual responsibility. LA’s reflection offers guidance here. She did not experience personal QI as additional burden—she experienced it as relief from the burden of being unheard and powerless. The tools reduced overwhelm by making complexity manageable. They did not replace professional support; they enabled more effective collaboration with professionals. The framework positions personal QI as enhancing agency and partnership, not as shifting responsibility for health outcomes entirely to individuals. Systemic factors, social determinants and resource constraints—these remain crucial. Personal QI works within contexts, not as a substitute for addressing those contexts.

## Broader implications and call to collaborate

The implications extend far beyond improved health outcomes, aligning with broader healthcare transformations. Systems are globally shifting from treatment-focused to prevention-oriented models, recognising that individuals managing health upstream prevents costly downstream interventions.^21^ LA’s experience illustrates this vividly: 3 years in secure hospital settings costing the NHS thousands of pounds could potentially have been prevented had she acquired systematic self-management capabilities earlier in her journey.

But prevention requires more than information—it requires capability. When individuals can systematically test what works for their specific contexts, prevention becomes personalised and sustainable. This applies to citizens broadly, including health and care staff managing their own well-being alongside demanding professional responsibilities.

For patients with chronic conditions, clinicians’ roles can shift from problem-solvers to guides and enablers, finding renewed purpose in supporting partnership rather than delivering top-down treatment. LA’s experience demonstrates this transformation: her GP and gynaecologist did not do less—they did different work, responding to evidence she brought, collaborating to interpret patterns and providing expertise within a more balanced partnership.

This shift supports people to be active partners in their own health and care, not passive observers or recipients.^9^ It may also help address clinician burnout by creating more satisfying, collaborative relationships.

This viewpoint is an explicit invitation to all stakeholders to collaborate in developing, refining and rigorously evaluating person-centred continuous improvement approaches.

*Researchers and innovators*: partner in codeveloping and evaluating personal QI frameworks, grounded in lived experience like LA’s, exploring their impact across diverse populations and health conditions.

*Healthcare systems and leaders*: champion equipping people with QI skills for both care delivery and personal well-being. The infrastructure for organisational QI already exists in many systems; extending it to personal QI requires relatively modest additional investment for potentially substantial returns.

*People with lived experience*: partners in this journey. Share your stories and expertise—your knowledge is the foundation on which successful personal QI approaches must be built.^5^ LA’s insights about what makes QI tools accessible, how they create hope, why visibility matters and when measurement helps versus hinders—this wisdom cannot be derived from theory alone.

*Clinicians and peer supporters*: you are ideally positioned to support personal QI development. You already engage in collaborative goal setting, problem solving and systematic thinking with patients. How might your practice evolve if individuals you support had structured tools for generating evidence about their own experience?

## Conclusion: QI as a way of being

The ultimate aim extends beyond better self-management, towards cultivating *QI as a way of being*—a fundamental life skill. When individuals are equipped with these tools and habits for systematic health improvement, they engage with ongoing learning and adaptation, no longer passive recipients of care but active authors of their own health stories.

LA’s reflection on hope captures this transformation: “Even when things didn’t work, I felt empowered because I could see what I was working on. It gave me hope.” Hope here is not wishful thinking—it is grounded in capability, in systematic inquiry and in the confidence that comes from being able to test, learn and adapt.

Her insight brings us full circle: ‘they can argue with what they listen to, but they cannot argue with what they see’. QI made the invisible visible, transforming subjective experience into shareable evidence, shifting power dynamics from dismissal to collaboration, replacing overwhelm with manageability and, perhaps most importantly, cultivating hope in circumstances where it had repeatedly been crushed.

CQI continues to transform how healthcare systems learn and adapt.^22^ The next chapter begins when every person can become a systematic improver of their own well-being—when the tools that revolutionised organisational learning become the instruments of personal agency, partnership and hope.

## Supplementary material

10.1136/bmjoq-2025-003812 online supplemental file 1
