# Supplementary material for: Quality improvement for self-management: the DIALOG+QI framework
Source: BMJ Open Qual. 2026 Jan 22;15(1):e003812. doi: 10.1136/bmjoq-2025-003812 (PMC12829366; doi:10.1136/bmjoq-2025-003812)
Supplement: online supplemental file 1 [file bmjoq-15-1-s001.pdf]

# CONCEPTUAL MODEL:
# INTEGRATING DIALOG+ AND QI

## A. DIALOG+ (current model): Measurement System

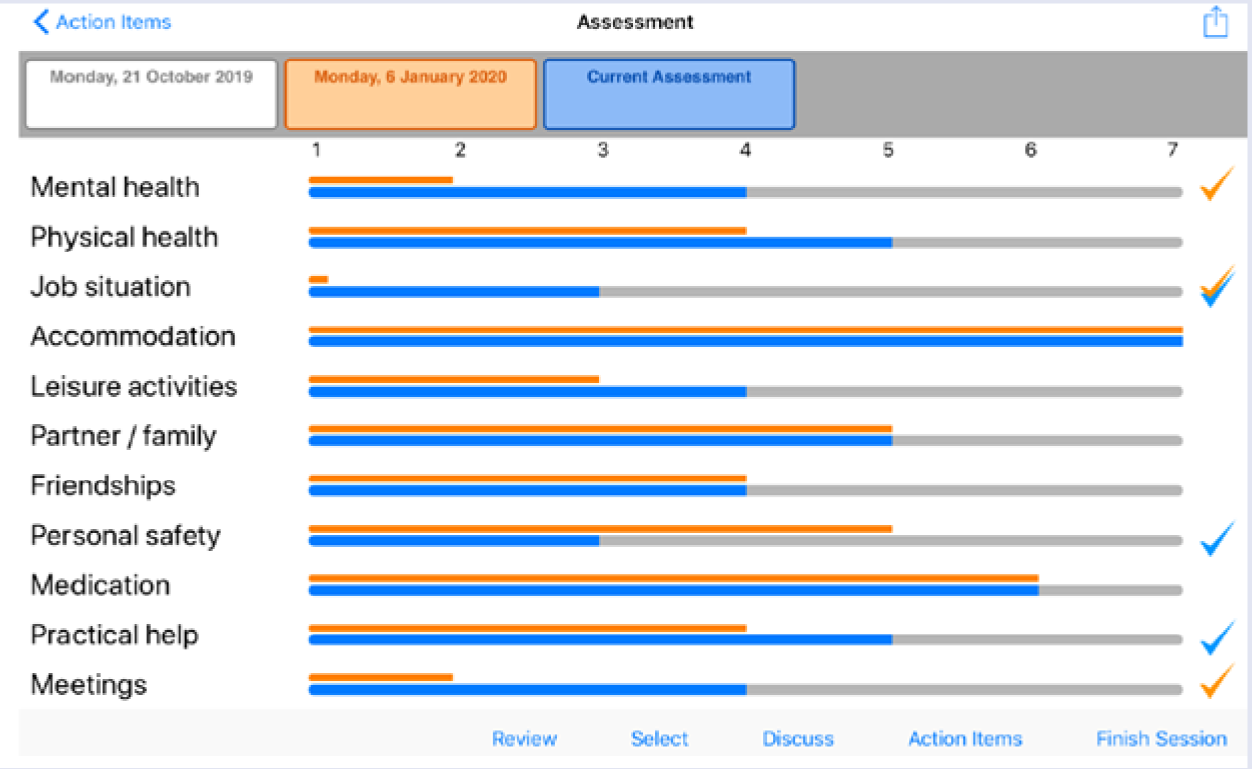


## B. DIALOG+QI (concept): Driver Diagram

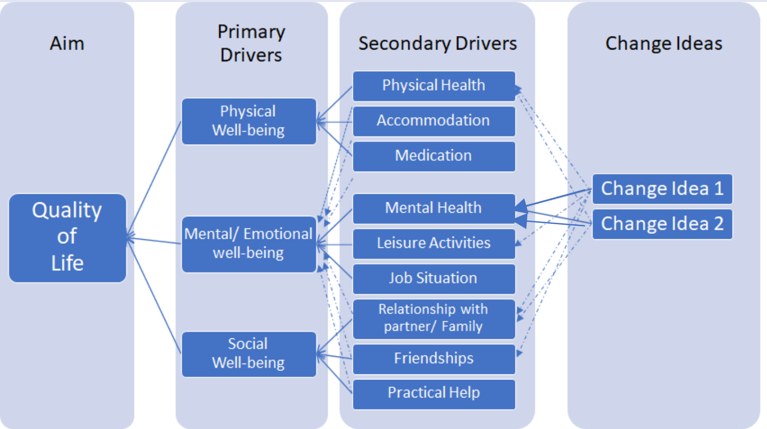


## C. Tracking Progress: Run Charts for DIALOG+ Domains

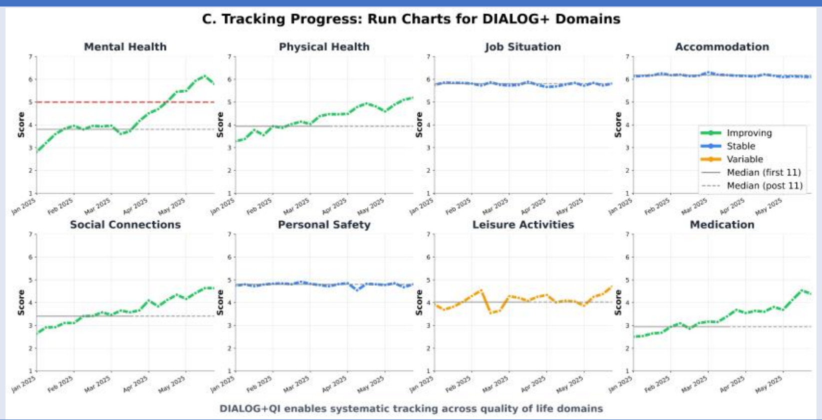


## D. Maria's Journey: Outcomes and Process Measures

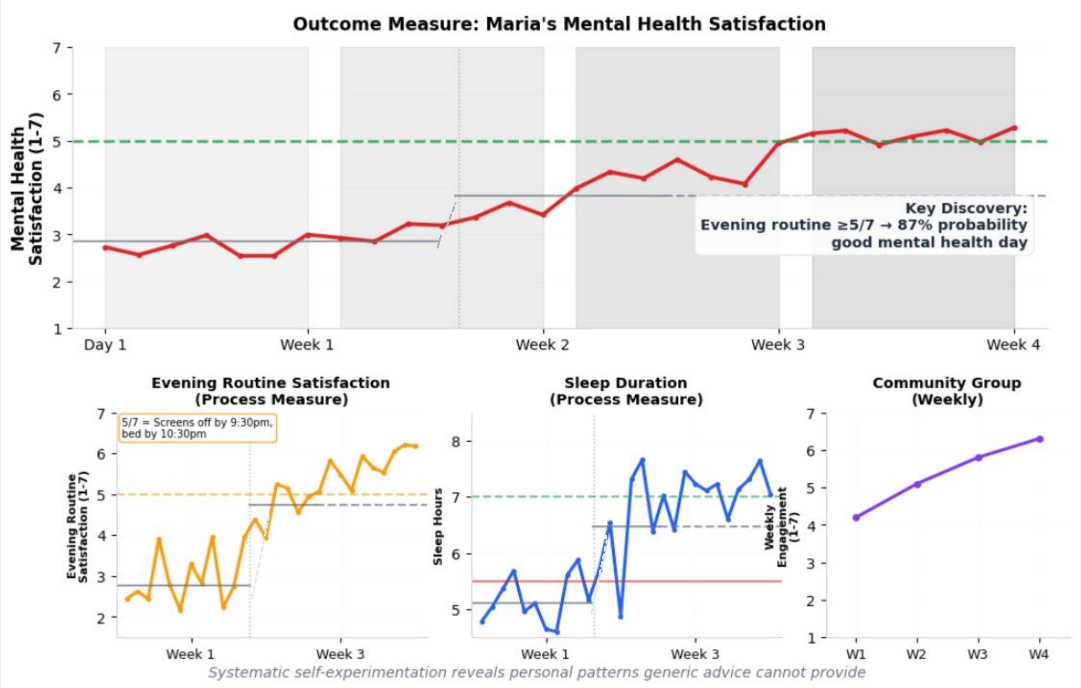


***Figure 1.* Conceptual Model for DIALOG+QI**
***A:*** *DIALOG+ (current practice): Routine outcome measurement across life and care domains.*
***B:*** *Driver diagram illustrating how DIALOG+ domains structure improvement efforts through primary and secondary drivers, linked to targeted change ideas.*
***C:*** *Example run charts tracking progress in individual DIALOG+ domains over time.*
***D:*** *Visualisation of an individual patient's journey (Maria), combining outcome and process measures to monitor improvement in real-world practice.*
